# Serum neurofilament light chain levels as a biomarker of neuroaxonal injury and severity of oxaliplatin-induced peripheral neuropathy

**DOI:** 10.1038/s41598-020-64511-5

**Published:** 2020-05-14

**Authors:** Su-Hyun Kim, Moon Ki Choi, Na Young Park, Jae-Won Hyun, Min Young Lee, Ho Jin Kim, Su Kyung Jung, Yongjun Cha

**Affiliations:** 1 Department of Neurology, Research Institute and Hospital of National Cancer Center, Goyang, Korea; 2 Center for Colorectal Cancer, Research Institute and Hospital of National Cancer Center, Goyang, Korea; 3 Eye Clinic Hospital, Research Institute and Hospital of National Cancer Center, Goyang, Korea

**Keywords:** Cancer, Biomarkers, Neurology, Oncology

## Abstract

We set out to determine the usability of serum neurofilament light chain (sNfL), serum glial fibrillary acidic protein (sGFAP), and retinal parameters by using optical coherence tomography (OCT) as reliable biomarkers of the progression of oxaliplatin-induced peripheral neuropathy (OIPN). Forty-three patients scheduled to undergo oxaliplatin-based chemotherapy at the National Cancer Center of Korea between June 2018 and October 2019 were prospectively assessed at baseline, 3 months, and 6 months of chemotherapy. Patients were assessed on clinical scales and underwent OCT, sNfL, and sGFAP level measurement at each follow-up visit. By applying the National Cancer Institute-Common Toxicity Criteria (NCI-CTC), OIPN was classified as grade 1 in 12 (28%) patients, grade 2 in 25 (58%), and grade 3 in 5 (12%) at 6 months of chemotherapy. sNfL levels increased during oxaliplatin administration, while serial sGFAP levels or retinal parameters did not change. Patients with grade-3 OIPN showed significantly higher mean sNfL levels than patients with grade 0–2 OIPN at 6 months of treatment. At 4–6 months after completion of chemotherapy, sNfL levels were significantly reduced compared to the levels at 6 months of chemotherapy. Monitoring of sNfL during chemotherapy can indicate ongoing neuroaxonal injury and the severity of OIPN.

## Introduction

Oxaliplatin is the principal chemotherapeutic agent for the treatment of colorectal cancer (CRC) and is also used in patients with pancreatic, gastric, and other cancers^1^. Although oxaliplatin has improved overall survival rates, the treatment leads to significant dose-dependent neuro-toxicities that can negatively impact the long-term quality of life of cancer survivors, because they may experience persistent symptoms even after the cessation of chemotherapy^2–4^.

There is currently a limited availability of diagnostic facilities for the objective evaluation of the severity of chemotherapy-induced peripheral neuropathy (CIPN). The assessment of CIPN usually depends on the patient’s symptoms that are judged according to physician evaluation scores such as the National Cancer Institute-Common Toxicity Criteria (NCI-CTC), which show poor sensitivity to change and low interrater reliability^5^. Patient-reported outcomes (PRO) have recently been recommended as a supplementary tool for the assessment of CIPN, but matched comparisons between PRO data and clinician-rated CIPN findings show only poor to modest agreement^6^, and their sensitivity to detect small or moderate differences in the severity of CIPN is unknown^7^. Because clinical assessments of CIPN usually depend on the patient’s subjective evaluation of their symptoms, objective and quantitative measures are fundamental. In clinical practice, quantitative methods such as nerve conduction studies (NCS) have been used. However, NCS are not widely used to evaluate patients with cancer in daily clinical practice, because they require referral to specialized neurological laboratories and often cause discomfort^8^. Therefore, a simple, easy-to-use method for the objective and quantitative assessment of CIPN needs to be established.

Neurofilament light chain (NfL) is a cytoskeleton protein expressed in large calibre myelinated axons^9^. Glial fibrillary acidic protein (GFAP) is a cytoskeletal protein expressed in Schwann cells, whose expression is increased when these cells lose contact with axons undergoing Wallerian degeneration^10^. Neuroaxonal damage in peripheral nerves results in the release of NfL and GFAP into the extracellular space and peripheral blood, depending on the extent of damage^9,11^. The emergence of the single molecule arrays (SIMOA), an ultra-sensitive enzyme-linked immunosorbent assay (ELISA) technique, for measuring NfL and GFAP levels in peripheral blood has led to the renaissance of NfL and GFAP as biomarkers in several diseases characterized by axonal loss in the central nervous system and peripheral nervous system, including multiple sclerosis, stroke, head injury, dementia, and immune-mediated neuropathy^9,11^. Optical coherence tomography (OCT) is a noninvasive method permitting precise measurements of the retinal layers including the retinal nerve fibre layer (RNFL). RNFL thickness in the retina can be used as a measurement of axonal loss in the anterior visual pathways. Using OCT, axonal loss, evident in the thinning of the RNFL, has been reported in a range of neurological disorders including amyotrophic lateral sclerosis^12^, Parkinson’s disease^13^, and diabetic peripheral neuropathy^14^.

In this study, we set out to determine the usability of serum NfL (sNfL), GFAP (sGFAP), and RNFL as reliable and easily accessible biomarkers of the progression and severity of chronic oxaliplatin-induced peripheral neuropathy (OIPN).

## Results

A total of 43 patients completed the serial evaluations during the 6 months of treatment. Dose reduction of oxaliplatin was performed in 30 (70%) patients; this was applied due to neurotoxicity in 5% of the patients. At 6 months of chemotherapy, chronic OIPN was present in 42 (98%) of 43 patients; classified as grade 1 in 12 (28%), grade 2 in 25 (58%), and grade 3 in five (12%) patients, respectively. At 3 months of chemotherapy, OIPN was present in 36 (84%) of 43 patients; classified as grade 1 in 30 (70%), and grade 2 in 6 (14%) patients, respectively. The demographic characteristics, cancer stage, cumulative dose of oxaliplatin, proportion of patients receiving a bevacizumab or cetuximab combination, and proportion of patients with diabetes did not statistically differ between patients with different OIPN grades at 6 months of treatment (Table 1).

**Table 1:** Clinical characteristics and neuropathy parameters of participants with chronic OIPN of grade 0–1, 2, and 3 at 6 months of treatment.

	Total (n = 43)	OIPN grade 0–1 (n = 13)	OIPN grade 2 (n = 25)	OIPN grade 3 (n = 5)	p-value
Age, years, mean (SD)	58.7 (9.1)	56 (12)	59 (7)	62 (7)	0.961
Sex, female, %	35	54	32	0	0.106
Colorectal cancer, %					0.466
Stage II/III/IV	9/75/16	0/77/23	16/72/12	0/80/20	
Cumulative dose of oxaliplatin, mg/m^2^, mean (SD)					
At 3 months	475 (37)	465 (41)	476 (37)	492 (16)	0.356
At 6 months	858 (129)	42 (139)	860 (125)	891 (136)	0.781
Diabetes mellitus, %	16	15	12	40	0.325
FOLFOX with bevacizumab or cetuximab, %	12	15	4	40	0.054
EORTC-CIPN20 at 3 months	24 (4)	22 (3)^c^	24 (4)	27 (4)^c^	**0.042**
Sensory	11 (2)	11 (2	11 (2)	12 (2)	0.124
Motor	9 (2)	8 (1)	9 (2)	10(1)	**0.02**
Autonomic	4 (1)	3 (1)	4 (1)	5 (2)	0.058
EORTC-CIPN20 at 6 months	24 (4)	22 (3)^c^	24 (4)^b^	27 (4)^bc^	**<0.001**
Sensory	14 (4)	12 (3)^c^	14 (3)^b^	19 (7)^bc^	**0.002**
Motor	14 (4)	10 (3)^c^	10 (2)^b^	17 (4)^b^	<**0.001**
Autonomic	4 (2)	4 (1)^c^	4 (2)	6 (2)^c^	**0.022**
a-SAP of median nerve (µV)	29 (14)	32 (17)	29 (13)	18 (4)	0.134
a-SAP of ulnar nerve (µV)	18 (9)	21 (9)	18 (9)	13 (2)	0.263
a-SAP of sural nerve (µV)	18(8)	21 (9)	18 (8)	13 (5)	0.215
SCV of median nerve (m/sec)	41 (6)	43 (5)	40 (6)	41 (6)	0.495
SCV of ulnar nerve (m/sec)	44 (4)	45 (2)	43(4)	47 (3)	0.242
SCV of sural nerve (m/sec)	38 (4)	40 (3)	37 (4)	39 (4)	0.225
NCS at 3 months					
a-SAP of median nerve (µV)	24 (12)	31 (14)	22 (10)	18 (4)	0.036
a-SAP of ulnar nerve (µV)	14 (6)	18 (5)	13 (6)	11 (2)	0.042
a-SAP of sural nerve (µV)	17 (8)	21 (9)	16 (7)	12 (5)	0.057
SCV of median nerve (m/sec)	39 (5)	39 (5)	38 (6)	37 (4)	0.697
SCV of ulnar nerve (m/sec)	42 (4)	43 (3)	40 (3)	41 (4)	0.14
SCV of sural nerve (m/sec)	17 (8)	21 (9)	16 (7)	12 (5)	0.176
NCS at 6 months					
a-SAP of median nerve (µV)	10 (7)	14 (8)^c^	9 (5)	3 (2)^c^	**0.003**
a-SAP of ulnar nerve (µV)	8 (5)	11(3)^ac^	8 (5)^a^	4(1)^c^	**0.002**
a-SAP of sural nerve (µV)	9 (4)	13 (4)^ac^	8 (4)^a^	6(3)^c^	**0.003**
SCV of median nerve (m/sec)	35(7)	37 (6)	35 (5)	28 (16)	0.067
SCV of ulnar nerve (m/sec)	36 (6)	39 (2)	35 (8)	38 (4)	0.156
SCV of sural nerve (m/sec)	33 (4)	34 (3)	32(4)	34 (3)	0.225

Abbreviations: FOLFOX, infusion of fluorouracil, leucovorin, and oxaliplatin; EORTC QLQ-CIPN20, European Organization for Research and Treatment of Cancer Quality of Life Questionnaire-Chemotherapy-Induced Peripheral Neuropathy 20 module; a-SAP, amplitude of sensory nerve action potential; SCV, sensory conduction velocity.

Data are mean ± SD or *n* (%) values. p-values <0.05 are shown in bold. ^a^p < 0.05 between patients with OIPN grade 0–1 and grade 2, ^b^p < 0.05 between patients with OIPN grade 2 and 3, ^c^p < 0.05 between patients with OIPN grade 0–1 and 3.

### Increase of sNfL levels over the course of treatment and severity of OIPN

**Figure 1:**
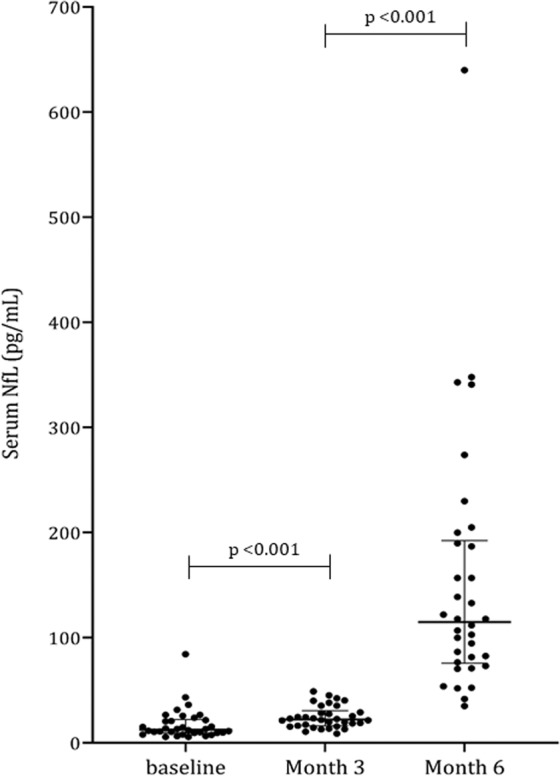
Serum neurofilament light chain levels over the course of treatment Data are median and interquartile ranges. p-values were calculated using the Wilcoxon signed rank test. NfL: neurofilament light chain.

**Figure 2:**
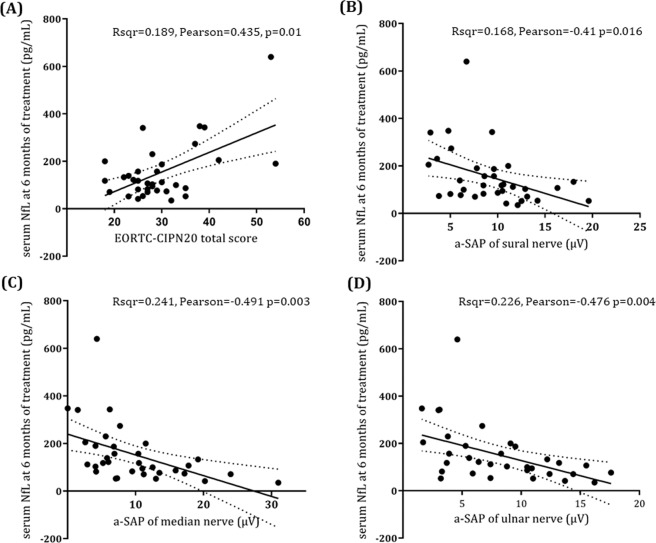
Relationship between serum neurofilament light chain levels at 6 months and main outcome measures. Sensory score of the EORTC QLQ-CIPN20 (**A)**, and a-SAP (µV) of the sural nerve (**B**), median nerve (**C**), and ulnar nerve (**D**). The scatterplot shows regression lines and 95% confidence intervals. sNfL: serum neurofilament light chain, EORTC QLQ-CIPN20: European Organization for Research and Treatment of Cancer Quality of Life Questionnaire-Chemotherapy-Induced Peripheral Neuropathy 20 module.

**Figure 3:**
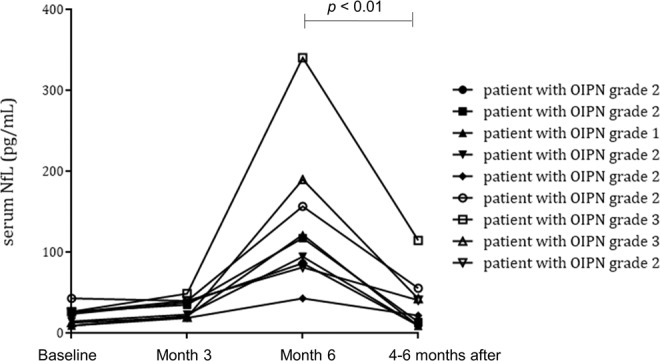
Serum neurofilament light chain levels for nine patients over the course of 6 months of chemotherapy and 4–6 months after chemotherapy. *p*-values were calculated using the Wilcoxon signed rank test. NfL: neurofilament light chain.

Serial sNfL concentrations were measured in 34 patients including all five patients with grade-3 OIPN. sNfL was increased during the course of oxaliplatin administration; the increase in mean sNfL concentrations was mild between baseline (median 12.7 [IQR 9.5,22.3]) and 3 months of treatment (median 22.3 [IQR 16.2,30.8]) (p < 0.001), while the changes between 3 and 6 months were more prominent (median 115.0 [IQR 75.9, 192.5]) (p < 0.001) (Fig. 1). At 6 months of treatment, sNfL levels showed significant differences according to OIPN grades (Table 2) (eFig. 1). There was no difference in sNfL level at 3 months of treatment between patients with grade 3 OIPN and grade 0–2 OIPN at 6 months of treatment (Table 2). The ROC curve analysis showed that at a cut-off value of 195 (pg/mL) for sNfL levels at 6 months, the sensitivity and specificity for grade-3 OIPN reached 80% and 86.2%, respectively. At 6 months of treatment, sNfL levels were positively correlated with the total score (r = 0.565, p < 0.001) and the sensory score of the EORTC QLQ-CIPN20 (r = 0.435, p = 0.01), and negatively correlated with the amplitude of sensory nerve action potential (a-SAP) of the sural nerve (r = −0.410, p = 0.016), median nerve (r = −0.491, p = 0.003), and ulnar nerve (r = −0.476, p = 0.004) (Fig. 2). Additionally, the sNfL levels at 4–6 months after completion of 6-month chemotherapy measured in nine patients showed a significant reduction compared to the levels at 6 months of chemotherapy (Fig. 3).

**Table 2:** Serum neurofilament light chain levels at baseline, 3 months, and 6 months of treatment according to neuropathy grade at 6 months of treatment, adjusted for age.

	OIPN grade 0–1 (n = 10)		OIPN grade 2 (n = 19)		OIPN grade 3 (n = 5)		Post-hoc p-value (Bonferroni)		
	Adjusted mean	95% CI	Adjusted mean	95% CI	Adjusted mean	95% CI	OIPN gr 0–1 vs 2	OIPN gr 2 vs 3	OIPN gr 1 vs 3
sNfL at baseline (pg/mL)	14.1	4.6, 23.5	20.1	13.2, 26.9	16.1	2.7, 29.5	0.907	1.0	1.0
sNfL at 3 months (pg/mL)	21.2	15.2, 27.2	26.0	21.7, 30.4	25.8	17.4, 30.4	0.575	1.0	1.0
sNfL at 6 months (pg/mL)	91.6	38.9, 144.2	127.0	89.1, 165.0	373.4	298.8, 447.9	0.820	**<0.001**	**<0.001**

Abbreviations: OIPN, oxaliplatin-induced peripheral neuropathy; gr, grade; sNfL, serum neurofilament light chain; CI, confidence interval.

p-values <0.05 are shown in bold.

### Electrophysiological characteristics and EORTC-CIPN 20 scores in oxaliplatin treated patients

**Figure 4:**
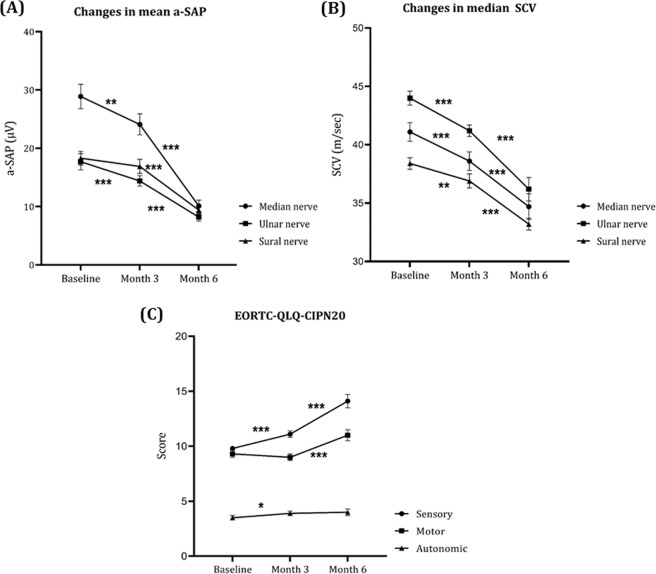
Amplitude of sensory nerve action potentials (a-SAPs) (**A**), sensory conduction velocity (SCV) (**B**), and scores on the EORTC QLQ-CIPN20 (**B**). Data are means and standard error values. EORTC QLQ-CIPN20: European Organization for Research and Treatment of Cancer Quality of Life Questionnaire-Chemotherapy-Induced Peripheral Neuropathy 20 module, OIPN: oxaliplatin-induced peripheral neuropathy, *p < 0.5; **p < 0.01; ***p < 0.001.

Of the 43 patients, a significant reduction in the a-SAP of the median and ulnar nerve was found at 3 months of treatment and changes in the a-SAP of the median, ulnar, and sural nerve were more pronounced at 6 months of treatment (Fig. 4A). Significant reductions of the sensory conduction velocity (SCV) in the median, ulnar, and sural nerves were also observed over the treatment course (Fig. 4B). However, no significant differences in SCVs were found between patients with different OIPN grades at 6 months of treatment (Table 1). At 6 months of treatment, patients with grade-3 OIPN showed a significant reduction in the a-SAP of the median, ulnar, and sural nerve compared to patients with grade-0–1 OIPN (Table 1). In addition, patients with grade-2 OIPN at 6 months of treatment showed a significant reduction in the a-SAP of the sural and ulnar nerve compared to patients with grade-0–1 OIPN (Table 1). Sensory, motor, and autonomic EORTC QLQ-CIPN20 scores increased with repeated cycles of treatment (Fig. 1B). At 6 months of treatment, patients with grade-3 OIPN showed higher sensory, motor, and autonomic scores than patients with grade-0–1 OIPN (Table 1).

### No change of retinal thickness or sGFAP levels in oxaliplatin treated patients

The serial full retinal thickness and inner retinal thickness parameters in all patients using OCT did not significantly change during the 6 months of chemotherapy (eFig. 2). As a preliminary investigation, serial sGFAP concentrations were measured in 10 patients, including two with grade-3 OIPN; there was no change in serial sGFAP levels during chemotherapy (Fig. 1). We therefore did not increase the number of patients included in the measurement of sGFAP levels.

## Discussion

We demonstrate a progressive increase in serum NfL levels in patients with OIPN undergoing chemotherapy that is closely related to clinically assessed patient-reported outcomes (EORTC QLQ-CIPN20) as well as physician-rated parameters (NCI-CTC). The relation between neuronal damage and sNfL concentrations is also supported by the clear positive association between sNfL levels and changes in sensory nerve amplitudes. NfL is highly specific for neuronal cell damage and eventual neuronal cell death, thereby offering a key advantage over other possible biomarkers. However, the role of NfL as a biomarker of neuroaxonal damage in CIPN has not yet been investigated in cancer patients. In 2018, Meregelli et al. reported an increase in blood NfL that was closely correlated with pathologically-confirmed axonopathy in vincristine-treated rats^15^. Oxaliplatin causes damage to the nuclei of dorsal root ganglions by forming adducts with nuclear and mitochondrial DNA, leading to neuronal apoptosis due to aberrant entry into the cell cycle, which corresponds to the pattern of neuronopathy^16^. The increase in serum NfL values in patients with OIPN and the significant correlation between serum NfL levels and disease severity support the role of NfL as a severity biomarker in OIPN.

In our cohort, the significant increase in sNfL levels between the 3- and 6-month evaluations, and the fact that no patient had grade-3 OIPN at 3 months, suggest that obvious neuronal damage occurs only after 3 months of oxaliplatin treatment. On the basis of a potential reduction in adverse events and non-inferiority in disease-free survival^4,17^, recent guidelines suggest that patients with low-risk stage III resected colon cancer should be offered 3 or 6 months of oxaliplatin-containing adjuvant chemotherapy after a discussion of the potential benefits and risks of harm associated with the different treatment durations^18^. Our study provides evidence that the serial monitoring of sNfL levels as a quantitative biomarker facilitates assessing the degree of ongoing neuroaxonal damage and tailoring the treatment schedule for the individual patient.

Clinical trials with neuroprotective agents have failed to show meaningful efficacy, perhaps because more sensitive and robust objective biomarkers are needed to detect changes. The usefulness of NCS in objectively assessing CIPN remains controversial. Although motor scores of EORTC-CIPN20 increased with repeated cycles of oxaliplatin treatment, no changes in compound muscle action potentials and motor conduction velocities of the motor nerves were found after treatment in our previous study^19^. Motor nerve study using NCS may not be sensitive enough to detect the subtle, gradual changes of motor nerve in OIPN patients. Previously we found a significant longitudinal decrease in the a-SAPs and SCVs of all examined sensory nerves during oxaliplatin-based chemotherapy^19^. Early amplitude decreases in the a-SAPs of sensory nerves have also been suggested as a predictive factor of severe OIPN^19,20^. However, the severity of clinical neuropathy does not always correlate to NCS findings. NCS can detect large fibre damage but are insensitive to changes in small diameter nerve fibres. In our previous study, although 50% of patients had grade-2 or grade-3 OIPN at 6 months of oxaliplatin treatment, abnormalities in the a-SAP of the sural nerve compared with normative age-matched controls were found in only 14% of patients^19^.

In this study, we found that all five patients with grade-3 OIPN showed significantly higher levels of sNfL than patients with grade-0–2 OIPN (sensitivity 80% and specificity 86% at cut-off level 195 pg/mL). Measuring sNfL levels to monitor the severity of OIPN may have an important advantage over NCS. Blood testing is routinely performed in patients with cancer undergoing chemotherapy, but NCS are not easily applicable in daily clinical practice. Most importantly, sNfL levels are thought to result from ongoing neuroaxonal damage, while NCS predominantly identify already acquired axonal damage. Significant reductions in sNfL levels were found at 4–6 months after the completion of chemotherapy in this study, while no significant recovery of the a-SAP of the sensory nerve was found even 6 months after the end of treatment in our previous study^19^. Ideally, the gold standard outcome measures for clinical trials should be reliable and valid, but also responsive to changes in neurological examinations/symptoms of the patient^21^. Therefore, the serial monitoring of sNfL levels during chemotherapy could be a surrogate endpoint (treatment target) in future trials on neuroprotective properties of treatment strategies.

A part of the increased sNfL in our patients may have originated from CNS neurotoxicity after chemotherapy. However, while previous studies in patients with CNS damage such as neurodegenerative dementia or multiple sclerosis reported an average sNfL level of 40–80 pg/mL^22–24^, our patients with severe OIPN had high sNfL levels (mean 373.4 pg/mL) at the end of chemotherapy. In CNS diseases, axonal injury results in NfL leakage into the extracellular space. These then diffuse into the cerebrospinal fluid and are transported into the peripheral blood. The blood NfL was more than 50-fold lower than CSF; therefore, the fact that patients with severe CIPN had higher sNfL levels than those in CNS diseases suggests that the majority of the sNfL originates from peripheral nervous system injury.

In animals treated with oxaliplatin, increased numbers of GFAP (astrocyte) immune-positive cells and enhanced function of the spinal astrocyte gap junction were reported^25,26^. However, our preliminary data in 10 patients did not show any association between sGFAP levels and severity of OIPN or chemotherapy progression. In addition, reductions in full and inner retinal thickness assessed using OCT were not significant during oxaliplatin-based chemotherapy, either because there is no significant change in retinal thickness induced by oxaliplatin or because conventional OCT is not sensitive enough to detect changes in retinal thickness during chemotherapy.

This study has several limitations. The main limitation is the small sample size. The number of patients with grade-3 OIPN is small to draw firm conclusions. Furthermore, we measured sNfL only at two timepoints during chemotherapy, and sNfL levels at 3 months did not predict the risk of grade-3 OIPN at the end of the treatment. The optimal timing and cut-off levels for sNfL to predict severe OIPN need to be serially assessed in future studies, ideally at every cycle. Finally, to further evaluate the prognostic potential of sNfL levels, a long-term follow-up for OIPN after the completion of chemotherapy is also required.

We propose sNfL as a marker of neuroaxonal damage and severity in OIPN. Monitoring of sNfL levels during chemotherapy is feasible, allows quantitative and serial measurements with minimal burden on the patient, and enables the monitoring of ongoing neuronal injury in real time. Extensive validation studies with substantially higher numbers of patients are needed to establish the value of these finding for clinical trials as well as for the general practice.

## Methods

### Patients

The Institutional Review Board of the National Cancer Center (NCC) of Korea approved this protocol (approval number: NCC-2018-0114), and data was anonymized to protect the identities of all subjects involved in this study. Written informed consent was obtained from all patients prior to study entry and all research was performed in accordance with relevant guidelines/ regulations. We prospectively studied patients with CRC who were scheduled to undergo oxaliplatin-based chemotherapy between June 2018 and October 2019 at the NCC. The inclusion criteria were 1) histologically confirmed CRC, 2) no prior chemotherapy for other types of cancer, and 3) adequate haematological, hepatic, and renal functioning. The exclusion criteria included a history of peripheral neuropathy, glaucoma, best corrected visual acuity <20/40, obvious macular disease, or alcohol abuse, prior exposure to neurotoxic agents, degenerative neurological disorders, and HIV. Seven patients who had well-controlled diabetes without neuropathy were included. Among the 58 patients who were initially registered in this study, 15 were excluded for reasons of consent withdrawal (*n* = 7), discontinuation of treatment due to transfer to another hospital (*n* = 2), treatment intolerance (nausea, general weakness, or neutropenia) (n = 4), or initiation of other chemotherapy because of disease progression (*n* = 2). No patients discontinued treatment because of OIPN.

### Chemotherapy regimen and dose modification

All patients were treated with preplanned 6 months of oxaliplatin-based chemotherapy with a modified FOLFOX6 (mFOLFOX6) regimen. The regimen consists of oxaliplatin, 85 mg/m^2^, concurrent with leucovorin 400 mg/m^2^, given as a 2-hour intravenous infusion on day 1, followed by a bolus 5-FU, 400 mg/m^2^ on day 1; and a continuous 5-FU, 1,200 mg/m^2^/day on day 1 and 2 (46-hour continuous infusion of a total 5-FU of 2,400 mg/m^2^)^27^. For patients, mFOLFOX6 was given every two weeks up to 12 cycles (6 months). Among seven patients with metastatic CRC, bevacizumab (*n* = 3) or cetuximab (*n* = 2) were given in combination with mFOLFOX 6^28,29^. Oxaliplatin dosing was modified according to the predefined, toxicity-based guidelines of Center for Colorectal Cancer of NCC (supplementary material 1). The oxaliplatin dose was reduced by 20% in the presence of persistent or transient (lasting for at least 14 days) painful paraesthesia, dysesthesia, or functional impairment. Along with oxaliplatin dose reductions, we reduced bolus and infusion doses of 5-FU by 20% in the event of grade-3/4 neutropenia or thrombocytopenia (or both), stomatitis, diarrhoea, or other grade-3 drug-related adverse effects. If grade-3 neurotoxicity persisted despite the 20% reduction in the oxaliplatin dose, subsequent cycles omitted the chemotherapy. Analgesics with or without antiepileptics could be administered to those with severe OIPN at the discretion of the investigators.

### Evaluation of peripheral neuropathy

All subjects underwent clinical examinations, NCS, OCT, and sampling of blood at baseline, 3 months, and 6 months of chemotherapy. OIPN was defined as a clinical syndrome characterized by persistent, symmetrical distal painful or nonpainful paraesthesia and dysesthesia^30^. The incidence and severity of OIPN were graded using version 3.0 of the National Cancer Institute-Common Toxicity Criteria (NCI-CTCv3), with severity classified as grade 1, 2, or 3^31^ by one neurologist (author S.H.K) and one oncologist (author M.K.C or Y.C), with agreement at every evaluation. Patient-reported outcomes were also used to quantify the frequency and severity of OIPN, using the EORTC QLQ-Chemotherapy-Induced Peripheral Neuropathy 20 module (EORTC QLQ-CIPN20)^32,33^. The NCS included analyses of sensory conduction in the median, ulnar (orthodromic technique), and sural (antidromic technique) nerves, with measurements of the peak-to-peak a-SAP and the SCV. We serially measured the concentrations of sNfL and sGFAP at baseline, 3 months, and 6 months of chemotherapy in 34 and 10 patients, respectively. The analyses were performed using an in-house assay on the single molecule array platform (SIMOA; Quanterix, Lexington, MA, USA) Detailed instructions can be found in the Simoa Homebrew Assay Development Guide (Quanterix). The intra-assay and inter-assay coefficients of variation were <10%. We additionally measured sNfL levels at 4–6 months after completion of 6-month chemotherapy in nine patients who started the chemotherapy during the early phase of this study. OCT examination using spectral domain OCT (3D OCT-1 Maestro; Topcon corp. Tokyo, Japan) were performed at every visit. The 3D OCT measured a 6×6-mm area that was centred on the fovea using built-in software. The average macular RNFL, the ganglion cell layer + inner plexiform layer (GCL+) thickness, and the RNFL + GCL + IPL (GCL + + ) thickness in the superior and inferior hemiretina, and overall was calculated. In addition to the macular area, scans were acquired at 3.45 mm diameter centred at the optic nerve head and the peripapillary RNFL thickness was quantified. Through this optic disc scan, the overall RNFL thickness (RNFL overall) and the average of all quadrants; RNFL thickness in the superior, nasal, inferior, and temporal quadrants was measured.

### Statistical analysis

The normality of data distribution was assessed using the Kolmogorov–Smirnov test. The EORTC QLQ-CIPN20, sNfL, sGFAP, OCT, and NCS results were compared between serial assessments using the Friedman test of repeated measures one-way analysis of variance (ANOVA) and the Wilcoxon signed rank test for post hoc analysis. The results are expressed as means and standard errors or medians and interquartile ranges (IQRs). Clinical data and neuropathy parameters were compared across three OIPN severity groups (grade 0–1, grade 2, and grade 3) at 6 months of treatment. For categorical variables, we used a χ2 test or Fisher’s exact test. For continuous variables, we performed ANOVA and independent t tests for post hoc analysis, or the Kruskal–Wallis test and the Mann–Whitney U test. Comparisons of serum NfL levels were conducted using analysis of covariance (ANCOVA) with age as a covariate, and Bonferroni tests were used for post-hoc comparisons. Correlations between serum NfL levels and clinical data were analysed using Pearson’s correlation analysis. Receiver operating characteristics (ROC) analyses were performed to estimate the optimal cut-off level for sNfL with respect to the grade-3 neuropathy at 6 months of treatment. SAS software (version 9.3, SAS Institute, Cary, NC, USA) was used for all analyses. Two-sided p-value < 0.05 was considered statistically significant.

## Supplementary information

Supplementary Information.

Supplementary Information 2.

Supplementary Information 3.

## Data Availability

The datasets generated during and/or analysed during the current study are available from the corresponding author on reasonable request.
