# Supplementary material for: Serum neurofilament light chain levels as a biomarker of neuroaxonal injury and severity of oxaliplatin-induced peripheral neuropathy
Source: Sci Rep. 2020 May 14;10:7995. doi: 10.1038/s41598-020-64511-5 (PMC7224372; doi:10.1038/s41598-020-64511-5)
Supplement: Supplementary file 1 — Supplementary Information. [file 41598_2020_64511_MOESM1_ESM.docx]

**mFOLFOX6 dose modification guidelines of Center for Colorectal Cancer, National Cancer Center**

- mFOLFOX6 dose levels

| Drug | Initial | -1 dose level | -2 dose level |
| --- | --- | --- | --- |
| Oxaliplatin | 85mg/m2 | 65mg/m2 | 50mg/m2 |
| 5-fluorouracil (bolus) | 400mg/m2 | 320mg/m2 | 240mg/m2 |
| 5-fluorouracil (continuous infusion) | 2,400mg/m2 | 2,000mg/m2 | 1,600mg/m2 |

- Oxaliplatin dose modifications for oxaliplatin induced peripheral neurotoxicity (OIPN)

| NCI-CTCAE v3.0 | Duration | |
| --- | --- | --- |
|  | 1-7 days | Persistent |
| Grade 1 | None | None |
| Grade 2 | None | -1 dose level |
| Grade 3 | First occurrence: -1 dose level  Second occurrence: -2 dose level | Discontinue |
| Grade 4 | Discontinue | Discontinue |

- Dose modification for neutropenia

| NCI-CTCAE v3.0 | Oxaliplatin | 5-fluorouracil |
| --- | --- | --- |
| Grade 1 | None | None |
| Grade 2 | None | None |
| Grade 3 | First occurrence: -1 dose level  Second occurrence: -2 dose level | None |
| Grade 4 | First occurrence: -1 dose level  Second occurrence: -2 dose level | First occurrence: -1 dose level  Second occurrence: -2 dose level |

- Dose modification for thrombocytopenia

| NCI-CTCAE v3.0 | Oxaliplatin | 5-fluorouracil |
| --- | --- | --- |
| Grade 1 | None | None |
| Grade 2 | None | None |
| Grade 3 | First occurrence: -1 dose level  Second occurrence: -2 dose level | None |
| Grade 4 | First occurrence: -2 dose level  Second occurrence: discontinue | None |
