# Supplementary figures and images for: Serum neurofilament light chain levels as a biomarker of neuroaxonal injury and severity of oxaliplatin-induced peripheral neuropathy
Source: Sci Rep. 2020 May 14;10:7995. doi: 10.1038/s41598-020-64511-5 (PMC7224372; doi:10.1038/s41598-020-64511-5)

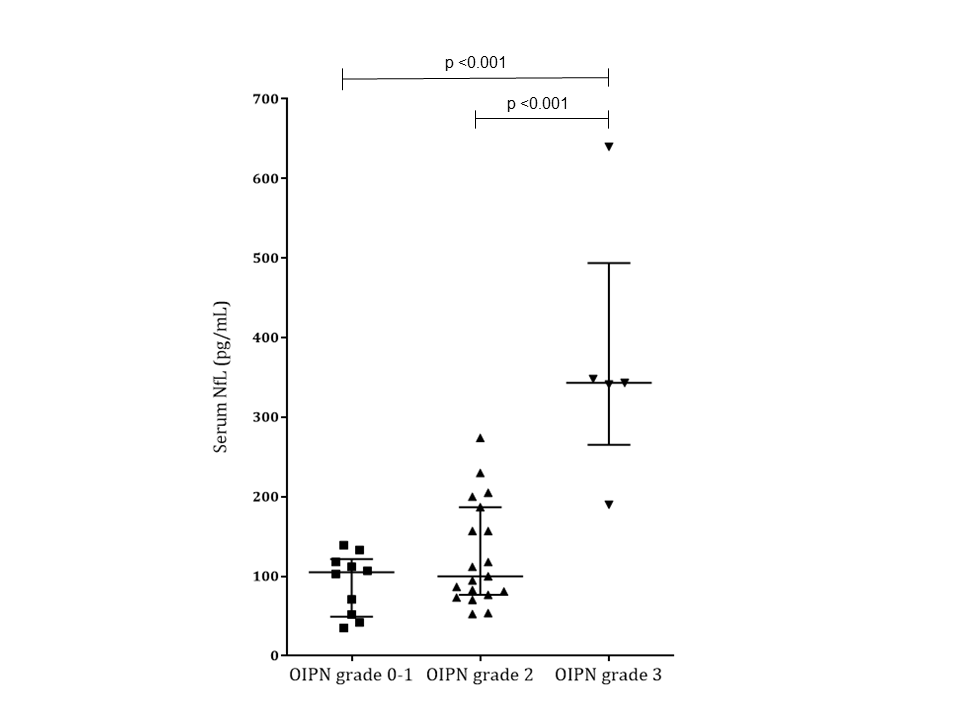

Supplement: Supplementary file 2 — Supplementary Information 2. [file 41598_2020_64511_MOESM2_ESM.tif]

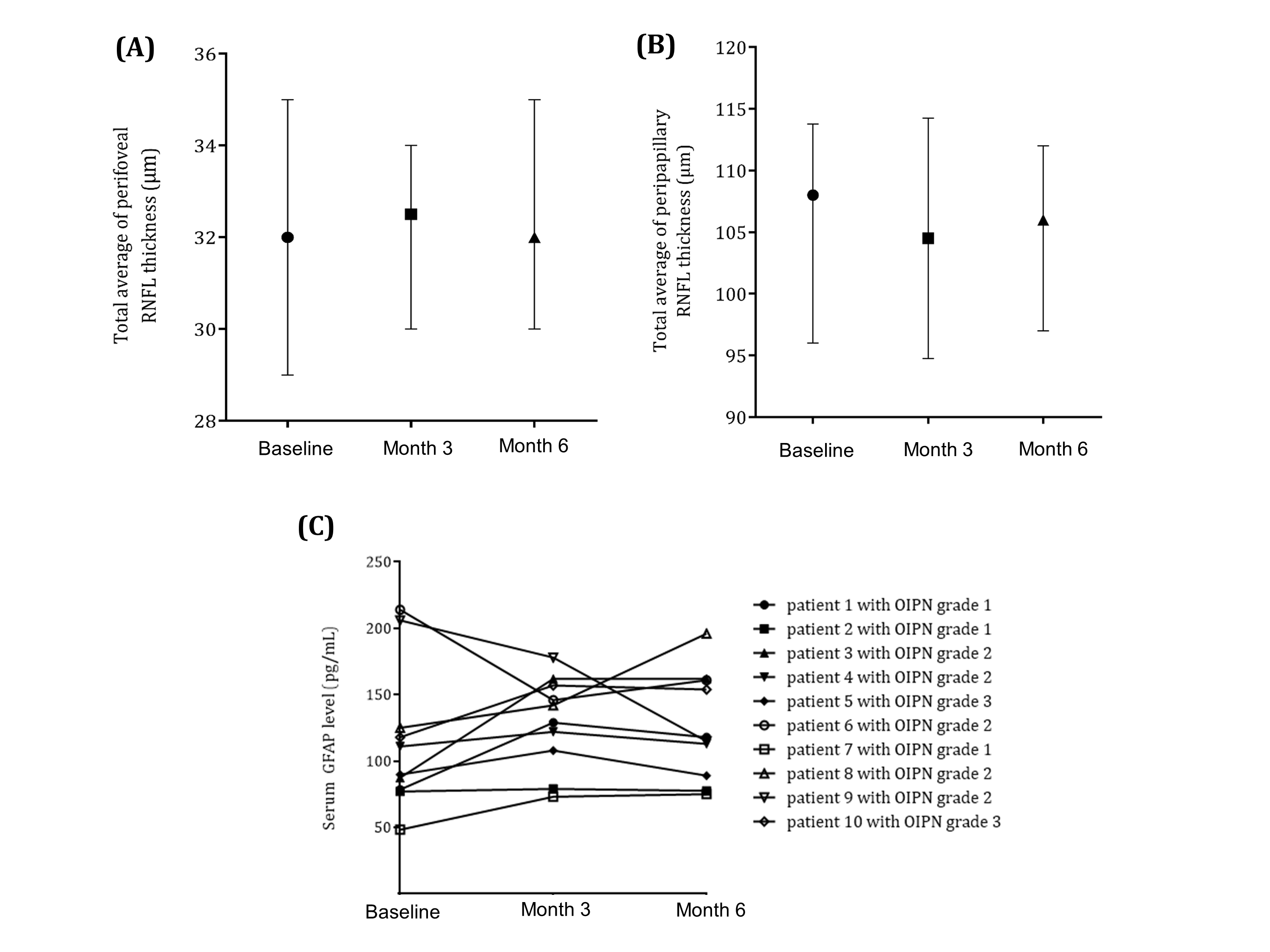

Supplement: Supplementary file 3 — Supplementary Information 3. [file 41598_2020_64511_MOESM3_ESM.tif]
